# Factors that influence the outcome of open urethroplasty for pelvis fracture urethral defect (PFUD): an observational study from a single high-volume tertiary care center

**DOI:** 10.1007/s00345-015-1533-4

**Published:** 2015-03-14

**Authors:** Qiang Fu, Yu-meng Zhang, Guido Barbagli, Jiong Zhang, Hong Xie, Ying-long Sa, San-bao Jin, Yue-min Xu

**Affiliations:** 1 Department of Urology, Shanghai Jiao Tong University Affiliated Sixth People’s Hospital, 600 Yishan Road, Shanghai, 200233 China; 2 Center for Urethral and Genitalia Reconstructive Surgery (GB, EP), Via dei Lecci 22, 52100 Arezzo, Italy

**Keywords:** Direct vision internal urethrotomy (DVIU), Pelvic fracture urethral injury (PFUI), Pelvic fracture urethral defects (PFUD), Urethroplasty

## Abstract

**Purpose:**

To report the clinical features of pelvic fracture urethral injury (PFUI) and assess the real effect of factors that are believed to have adverse effects on delayed urethroplasty.

**Methods:**

An observational descriptive study in a single urological center examined 376 male patients diagnosed with PFUI who underwent open urethroplasty from 2009 to 2013. Analyzed factors included patient age at the time of injury, etiology of PFUI, type of emergency treatment, concomitant injuries, length and position of stricture, type of urethroplasty and the outcome of surgery. Univariate and multivariate logistic regression analyses were applied, together with analytical statistic methods such as *t* test and Chi-square test.

**Results:**

The overall success rate of delayed urethroplasty was 80.6 %. Early realignment was associated with reduced stricture length and had beneficial effect on delayed surgery. Concomitant rectum rupture, strictures longer than 1.6 cm and strictures closer than 3 cm to the bladder neck were indicators of poor outcome. Age, type of injury, urethral fistula and bladder rupture were not significant predicators of surgery outcome. Failed direct vision internal urethrotomy and urethroplasty had no significant influence on salvage operation.

**Conclusions:**

The outcome of posterior urethroplasty is affected by multiple factors. Early realignment has beneficial effect; while the length and position of stricture and its distance to bladder neck plays the key role, rectum rupture at the time of injury is also an indicator of poor outcome. The effect of other factors seems insignificant.

## Introduction

The clinical characteristics of patients who present with pelvic fracture urethral injuries (PFUIs) are variable and depend on the cause and type of injury [1]. The methods most suitable for emergency surgery and delayed surgery of these distraction lesions are still debated in the current literature, and there are great differences in the procedures used in developing and developed countries [2–4]. Numerous factors may negatively impact the success rate of delayed urethroplasty for pelvic fracture urethral defects (PFUDs), such as advanced age, type of injury, emergency treatment, associated injuries, stricture length and number of previous failed treatments [5, 6]. However, there is disagreement about the significance of these different factors. The low incidence of PFUI and the effect of underlying illness make it difficult to study the effect of different emergency treatment regimens.

China is a republic with 1.35 billion people. The rapid growth of the economy and of labor-intensive industries has contributed to increases in the number of traumatic injuries, including PFUIs and PFUDs [7]. Our center is the largest urethral referral center in China, and the large number of patients that we have treated allowed us to retrospectively analyze patient records to assess the factors that influence the outcome of surgery for PFUD in patients who suffered from PFUI.

This study is an observational study of the management of PFUD in a Chinese Urology Department at a high-volume tertiary care center that assessed factors associated with outcome following delayed urethroplasty.

## Patients and methods

This retrospective, observational study investigated the effect of emergency treatment of PFUI and delayed treatment of subsequent PFUD in a high-volume hospital in China from 2009 to 2013. Prior to study onset, the institutional review board authorized chart review for descriptive analysis. The cutoff date for analysis was May 30, 2014. We performed multiple searches in our electronic medical records system, and initially 810 medical records of PFUD patients were found. Patients with incomplete clinical data or imaging exams, those lost to follow-up or follow-up shorter than 12 months, those who did not receive operation and whose result could not be clearly defined were excluded. After exclusion, 376 patients were included in our study. The primary objective was to describe the effects of the following variables on outcome: patient age at time of injury, etiology of PFUI, concomitant injuries, type of emergency treatment for PFUI and type of delayed treatment of PFUD. A secondary end point was the outcome of delayed urethroplasty. Univariate and multivariate logistic regression were used to analyze the possible influence of these factors. We also utilized descriptive and analytical statistic methods such as Chi-square test and *t* test to describe the clinical pattern of these patients.

Patient records were reviewed and analyzed by one researcher. Radiographic images were examined by a different researcher who was an experienced imaging professional and blinded to patient records. Stricture length and position were measured using the combination of retrograde and voiding urethrography. Posterior urethral reconstruction was classified as a failure when: (1) recurrent stricture which could not pass a 16.2Fr flexible cystoscope; (2) postoperative urethrography indicating recurrent stricture; (3) obstructive symptoms requiring frequent dilation, self-catheterization or direct vision internal urethrotomy (DVIU); or (4) urinary retention that required suprapubic diversion. All patients were referred to our tertiary high-volume center, and stringent follow-up procedures were performed that included clinical and instrument assessment at 3 months after surgery and every 12 months thereafter.

## Results

### History and clinical pattern of patients

The patients’ history and clinical information are summarized in Table 1. Three hundred seventy-six male patients with a median age of 35.33 years (range 4–79 years) were included in the study. All of them had PFUI and posterior urethral stricture. Most of them had their pelvis fracture treated elsewhere, and we would wait for at least 3 months after the injure before they could receive urethroplasty. For patients with persistent infection or abscess, we would give them antibiotics treatment and debridement if necessary. They cannot receive urethroplasty until their infection was brought under control, and urine culture showed two consecutive negative results. Overall success rate was 80.6 %. In our study population, PFUI was rare in children (3.7 %) and adolescents (7.7 %). Most patients (85.1 %) were adults between 18 and 60 years old. Blunt low-speed crush injury and falling mass hit cause more rectal rupture than other types of injury (14 of 31 crush injuries and 11 of 53 falling mass hits, both *p* < 0.01). Infection–abscesses were present in 20 % of patients (3 of 15) who suffered from straddle injury, significantly more common than other injury types (*p* < 0.01). Suprapubic cystostomy was the most common emergency treatment (*n* = 186, 49.5 %), followed by emergency open realignment (*n* = 137, 36.4 %). Only four patients (1.06 %) received endoscopic realignment (ER). The success rate of delayed urethroplasty was highest after cystotomy (*n* = 186, 80.6 %) and open realignment (*n* = 137, 81.6 %). Early open repair had lowest success rate (*n* = 12, 66.7 %). To elucidate the effect of realignment, we excluded patients who had received open surgery (*n* = 97) before we compared the result of realignment and cystotomy. Our data showed that comparing with cystotomy, early open realignment contributed to shorter strictures (2.04 vs. 2.40 cm, *p* = 0.037) and did not influence delayed urethroplasty (80.4 vs. 78.4 %, *p* = 0.753).

**Table 1:** History and clinical data of patients enrolled

Variables	Overall	Success	Failure	*p*
No. of patients (%)	376	303 (80.6)	73 (19.4)	
Age (year)				0.045
Mean	35.33	36.07	32.23	
Range	4–79	4–79	5–65	
Etiology				0.335
Car accident (%)	219	176 (80.3)	43 (19.7)	
Crush^a^ (%)	31	22 (71.0)	9 (29.0)	
Fall from height (%)	53	48 (90.6)	5 (9.4)	
Fall astride (%)^c^	15	14 (93.3)	1 (6.7)	
Fall (%)	4	4 (100)	0 (0)	
Hit by falling object (%)	53	39 (73.6)	14 (26.4)	
Gun shot (%)	1	0 (0)	1 (100)	
Length of stricture (cm)	2.18	2.09	2.56	0.017
Position of stricture (cm)^b^	3.26	3.41	2.61	0.000
Concomitant injuries and comorbidities				
Bladder rupture (%)	22	16 (72.7)	6 (27.2)	0.398
Rectum rupture (%)	42	27 (64.3)	15 (35.7)	0.006
Fistula (%)	35	26 (74.3)	9 (25.7)	0.364
False passage (%)	8	7 (87.5)	1 (12.5)	1.000
Infection or abscess (%)	10	9 (90)	1 (10)	0.694
Emergency treatment				0.575
Cystostomy (%)	186	150 (80.6)	36 (19.4)	
Open realignment (%)	137	111 (81.0)	26 (19.0)	
Endoscopic realignment (%)	4	4 (100)	0 (0)	
Catheterization (%)	31	24 (77.4)	7 (22.6)	
Early repair (%)	12	8 (66.7)	4 (33.3)	
No surgery intervention (%)	6	6 (100)	0 (0)	
Previous failed urethroplasty				0.523
0 (%)	279	222 (79.6)	57 (20.4)	
1 (%)	85	70 (82.4)	15 (17.6)	
2 or more (%)	12	11 (91.7)	1 (8.3)	
Previous DVIU^d^				0.297
0 (%)	272	216 (79.4)	56 (20.6)	
1 (%)	75	65 (86.7)	10 (13.3)	
2 or more (%)	29	22 (75.9)	7 (24.1)	
Surgery type				0.007
Simple anastomosis (%)	126	113 (89.7)	13 (10.3)	
Crural separation (%)	131	104 (79.4)	27 (20.6)	
Pubectomy (%)	103	74 (71.8)	29 (28.1)	
Retrocrural rerouting (%)	3	2 (66.7)	1 (33.3)	
Transpubic repair (%)	2	0 (0)	2 (100)	
Other^e^ (%)	11	10 (90.9)	1 (9.1)	

^a^Low speed blunt crush injury

^b^This number indicates the distance between the bladder neck and the distal end of the stricture

^c^These patients fell from a height and straddled on hard objects, causing pubis rami fracture and posterior urethral injury at the same time

^d^ *DVIU* direct vision internal urethrotomy

^e^Including urethrostomy, penile flap, lingual flap, scrotal flap, bladder wall flap, pull-in surgery and penile transposition

A total of 164 patients had received urethroplasty or DVIU before they came to our center. DVIU has become a common procedure in China, with 104 patients receiving 146 DVIUs (1.40 per patient). Our data showed that one failed DVIU or urethroplasty does not significantly affect the salvage operation (*p* = 0.29 for failed DVIU and 0.52 for failed open surgery).

When planning surgery for a patient, stricture length and position are our first concern. The types of injury have no relation with our choice of surgery type (*p* = 0.37). We would choose the optimum type of surgery according to different conditions. We employed muscle flap or tissue flap to repair fistula; we used cystoscopy to identify real urethra from false passage; and we employed crural separation, pubectomy, retrocrural rerouting and transpubic repair to compensate long urethral defect. Long strictures or strictures close to the bladder neck often requires more complex surgery and has greater risk of failing. In Table 1, we can clearly see that the success rate decreases with the escalating complicity of surgery.

### Outcome predictors and risk analysis

Multiple factors are believed to influence the outcome of urethroplasty, so we applied multivariate analysis in order to control the influence of other factors and better reveal the real effect of each factor. Analyzed variables included age, length and position of stricture, emergency treatment, concomitant injuries, previous failed urethroplasty and DVIU. Table 2 summarizes the univariate and multivariate analyses of different factors.

**Table 2:** Univariate and multivariate analyses of different factors that may influence the outcome of urethroplasty

Factors	Univariate analysis			Multivariate analysis		
	*p*	OR	95 % CI of OR	*p*	OR	95 % CI of OR
Age	0.045	1.018	1.000–1.037	0.808	1.002	0.983–1.023
Length of stricture						
<1.6 cm	–	1.000 (Ref)		–	1.000 (Ref)	
>1.6 cm	0.001	2.828	1.534–5.214	0.046	1.974	1.011–3.855
Position of stricture^a^						
>3 cm	–	1.000 (Ref)		–	1.000 (Ref)	
<3 cm	0.000	5.235	3.001–9.131	0.000	4.127	2.238–7.612
Emergency treatment^b^						
Cystostomy	–	1.000 (Ref)		–	1.000 (Ref)	
Catheterization	0.834	0.942	0.538–1.649	0.986	1.005	0.539–1.874
Early repair	0.999	–	–	0.999	–	–
Open realignment	0.222	0.452	0.127–1.616	0.047	0.223	0.051–0.980
Concomitant injuries and comorbidities						
Bladder rupture	0.341	1.606	0.606–4.260	0.989	1.008	0.336–3.021
Rectum rupture	0.002	2.869	1.452–5.669	0.032	2.464	1.079–5.625
Infection or abscess	0.457	0.454	0.057–3.639	0.433	0.393	0.038–4.058
Fistula	0.186	1.691	0.776–3.685	0.577	1.311	0.506–3.396
Previous failed urethroplasty						
No	–	1.000 (Ref)		–	1.000 (Ref)	
1 or more	0.4	0.769	0.418–1.416	0.218	0.62	0.29–1.327
Previous failed DVIU^c^						
No	–	1.000 (Ref)		–	1.000 (Ref)	
1 or more	0.353	0.754	0.415–1.369	0.627	0.846	0.432–1.659

Emergency treatment “endoscopic realignment” and “no surgery intervention” and comorbidity “false passage” are not shown as their sample numbers are lower than 10

*OR* odds ratio, *CI* confidence interval

^a^The distance between the bladder neck and the distal end of the stricture

^b^Factor “endoscopic realignment” and “no surgical intervention” were not analyzed due to low sample number (both *n* < 10)

^c^Direct vision internal urethrotomy

Age seems not to be a significant risk factor [odds ratio (OR) 1.002, *p* = 0.808], although patients who failed urethroplasty were actually younger (see Table 1). Stricture longer than 1.6 cm is a significant predictor of failure, confirmed by both univariate and multivariate analyses (OR 1.974, *p* = 0.046), but the position of stricture is a more important predictor. In our data, patients with the proximal end of stricture closer than 3 cm to the bladder neck had success rate of 64.6 %, while patients with the distance >3 cm had 90.5 %. The multivariate analysis further confirmed its effect, as it has OR of 4.127. In univariate analysis, the open realignment did not show significance, but multivariate analysis revealed it to be a beneficial factor (OR 0.223, *p* = 0.047). There were only four patients who received endoscopic realignment (ER), so the effect of ER cannot be analyzed. Rectum rupture at the time of injury is a significant predictor of poor surgery outcome (OR 2.464, *p* = 0.032). Other concomitant injuries such as bladder rupture, fistula and persistent infection did not show significance. We were unable to analyze the effect of false passage due to low sample number (*n* = 8). Failed urethroplasty and failed DVIU seemed to have no significant influence on the salvage operation (*p* = 0.218 and 0.627, respectively).

## Discussion

Patients with PFUIs present with variable clinical characteristics, and multiple factors may influence the outcome of urethroplasty for PFUD. Some studies have found that a number of factors could influence the urethra repair, but most of them have relatively low sample amount, thus lack supporting evidence. Our hospital is the referral center for patients all over China, and the large sample amount could hopefully better reveal the real effect of these factors. Some of the results were unsurprising, but others were quite unexpected.

In our study, PFUI is more common in the 20- to 60-year age group, while children, adolescents and elder people have lower risk. Our data seems to be in contrast with surveys from other developing countries [2]. Many urologists believe that urethroplasty of children under 18 is more difficult and has worse outcome, but our data show that age is not a significant predictor of surgery outcome (OR 1.002, *p* = 0.808).

PFUI can be caused by various types of injury, and patients may present with diverse concomitant injuries including bladder rupture, rectal tear, splenic or hepatic rupture, rib fracture and pneumothorax [8]. Inappropriate treatment of PFUI can cause a variety of comorbidities. Local extravasation can cause persistent infection or even abscess, poorly managed rectum rupture can cause urethrorectal fistula, and urethral dilation and DVIU can cause false passage, and so on. Their influence on urethroplasty is largely unclear. As far as we know, our study is the first one to address the effect of these factors and to reveal the adverse effect of rectal rupture. Rectal rupture is often due to a significant traumatic force, and such injuries are more likely to create longer strictures. The contamination of fecal extravasation is another possible reason for the severe adverse effects of rectal rupture. Unlike the rectum, a bladder full of urine is more likely to rupture than an empty bladder under the same impact. This may explain why bladder rupture had no significant adverse effect on urethroplasty outcome. In our study, patients with urethral fistulas had lower success rates than those without these conditions, but this difference was not significant. Multivariate analysis further revealed that the presence of fistula would not significantly affect urethroplasty (*p* = 0.577, OR 1.311). Due to the relatively small number of cases, the effect of false passage (*n* = 8) and persistent infection (*n* = 10) on surgical outcome remains doubtful.

The emergency treatment of PFUI can be challenging. Urethral realignment, especially endoscopic realignment (ER), is widely used in developed countries. Numerous studies reported that ER may save about 30–70 % patents from urethroplasty and reduce the length of the subsequent stricture [9–15]. However, the possible influence of ER on delayed urethroplasty is unclear, because reports from different studies are contradictory [10, 13, 16]. Our study supports the beneficial effect of early realignment with high sample volume. It can reduce stricture length by about 0.4 cm and has no negative influence on delayed urethroplasty. We believe that this 0.4 cm could make some difference, as stricture length is a major predictor of surgery outcome. To our knowledge, this study has the largest number of patients among studies with similar purpose and could help clarifying the debate about realignment. Unfortunately, in our survey of 376 patients, only four patients underwent ER, so we are unable to draw any conclusion about the efficacy of ER. But as ER and open realignment are based on the same principle of joining the broken ends together, we could deduce that ER should have similar effect. Primary suturing of urethral injuries has largely been abandoned due to poor outcome, and our data showed that if it fails, it has the lowest success rate among all emergency treatments [17].

Length and position of stricture are two important predicators of urethroplasty. Stricture length has always been regarded as an important predictor of surgery outcome, as longer stricture requires more dissection, has poorer blood supply and contributes to higher tension. Our study further confirmed this opinion. However, comparing to stricture length, the position of stricture is a more significant predictor with OR of 4.127. Strictures close to the bladder neck usually have poor exposure when using transperineal approach and leave little space for instruments during the suturing of mucosa. It also requires more dissection of surrounding tissue to expose the proximal end of urethral. All of these increase the difficulty of operation and have negative impact on surgery outcome.

Our survey showed that one failed DVIU had no adverse effects on delayed urethroplasty. That means a single DVIU can be a worthy attempt to solve dysuria symptom after realignment or urethroplasty, which is minimally invasive and has little adverse effect. But the effect of repeated DVIU could not be analyzed due to low sample amount. It is generally agreed that a previous failed urethroplasty makes the subsequent operation more difficult [5, 16, 18, 19], and we hold the same opinion. Heavy fibrosis around the urethra, poor blood supply and the change of anatomical structure all makes the redo more challenging. But some studies showed that the subsequent operation had similar success as the initial surgery [16]. One study from our hospital showed that the subsequent operation had a lower success rate, but that the difference was not significant [20]. The data from this study have no overlay with the previous one and have similar result. That means that when strictly following the principle of complete dissection of scar tissue and tension-free anastomosis, previous failed patients could also heal well and achieve satisfactory voiding.

Our descriptive study has some important implications for the field of trauma and urethral reconstructive surgery. This study has relatively large sample amount and can provide more evidence for the emergency and delayed treatment of urethral injuries. It supports the beneficial effect of early realignment and confirms the adverse effect of rectum rupture, long stricture and stricture close to bladder neck. Also, this is the first study to document management of PFUIs and repair of PFUDs in a Chinese population and could serve as a foundation for larger and more rigorous studies. Nonetheless, as an observational study, there are certain weaknesses. First, the study population may not be representative of the entire Chinese population, as China is a large and populous country. Second, our study is a case series that aggregated individual cases who were initially treated elsewhere before admission to our hospital. Thus, complete histories of surgical interventions and details about their injuries could not be accurately obtained, and the impact of some factors could not be assessed. Third, we only included patients within the last 5 years and the follow-up time was relatively short, so we cannot make any conclusions about the long-term outcomes.

## Conclusions

Success rate of urethroplasty is influenced by multiple factors, but their significance is still debated. Utilizing higher sample amount, our study provided more evidence on those debated problems and further confirmed those “common beliefs” which we took for granted but lacked evidence. Our study supports the benefit of early realignment and proposes some influential factors such as concomitant rectum rupture, length of stricture and the position of stricture. Other factors such as bladder rupture, urethral fistula, failed urethroplasty or DVIU seems to have insignificant effect.

## Abbreviations

DVIU: Direct vision internal urethrotomy
PFUI: Pelvic fracture urethral injury
PFUD: Pelvic fracture urethral defects
ER: Endoscopic realignment
OR: Odds ratio
